# A hypersensitive response‐like lesion‐inducing protein modulates basal immunity against grey leaf spot in maize

**DOI:** 10.1111/pbi.70239

**Published:** 2025-07-21

**Authors:** Tao Zhong, Chenyu Guo, Suining Deng, Mang Zhu, QianQian Zhang, Mingliang Xu

**Affiliations:** ^1^ State Key Laboratory of Plant Environmental Resilience China Agricultural University Beijing China; ^2^ National Maize Improvement Center, College of Agronomy and Biotechnology China Agricultural University Beijing China; ^3^ Center for Crop Functional Genomics and Molecular Breeding China Agricultural University Beijing China; ^4^ Department of Entomology and Plant Pathology North Carolina State University Raleigh NC USA

**Keywords:** maize, gray leaf spot, plant immunity, basal immunity, ROS

Crop diseases cause substantial yield losses, posing a significant threat to global food security (Savary *et al*., 2019). In maize, grey leaf spot (GLS), caused by the fungal pathogens *Cercospora zeina* and *Cercospora zeae‐maydis*, is a devastating foliar fungal disease capable of reducing yield by 11–69%, depending on the susceptibility of the hybrid cultivar and environmental conditions (Meisel *et al*., 2009; Mueller *et al*., 2020). Given the limitations of chemical control, genetic resistance is the most effective strategy for managing plant diseases, emphasizing the importance of elucidating the molecular mechanisms underlying pathogen resistance in maize.

Hypersensitive response (HR)‐like lesion‐inducing proteins (HRLs) are a group of proteins containing a conserved HR‐like lesion‐inducing domain, yet their functions and mechanisms in plant–pathogen interactions remain largely unknow. In rice, *OsHRL* expression is induced by *Xanthomonas oryzae* pv. *oryzae* (Xoo) infection, and its overexpression enhances resistance to *Xoo* (Park *et al*., 2010). In soybean, GmHRL1 interacts with the *Soybean mosaic virus* (SMV) P3 protein, facilitating its nuclear translocation (Luan *et al*., 2019). Additionally, in tobacco, *NbHRLI4* has been reported to induce cell death and negatively regulate *turnip mosaic virus* (TuMV) infection through a salicylic acid‐mediated pathway (Wu *et al*., 2021).

We recently cloned a major GLS resistance gene, *ZmWAKL*, encoding a cell wall‐associated receptor kinase‐like protein, and identified its co‐receptor ZmWIK via immunoprecipitation‐mass spectrometry (IP‐MS) (Zhong *et al*., 2024). To further explore the ZmWAKL immune complex, the resistant ZmWAKL^Y^ was screened against the cDNA library of the disease‐resistant inbred line Y32 using the split‐ubiquitin yeast two‐hybrid (Y2H) system. Fourteen proteins were identified as potential interactors, among which a HR‐like lesion‐inducing protein (ZmHRL, Zm00001d025020) was repeatedly detected (Table S1). Intriguingly, ZmHRL was not captured in our previous IP‐MS analysis. Yeast assays confirmed that most candidates, except for Zm00001d051405, bound to ZmWAKL^Y^ but showed weak or no interaction with ZmWAKL^Q^ (Figure 1a; Figure S1a). Transcriptome data (Yu *et al*., 2018) showed that *ZmHRL* was significantly induced by *C. zeina* infection (Figure S1b). Given the role of HRLs in plant disease resistance (Park *et al*., 2010; Wu *et al*., 2021), *ZmHRL* was selected for further investigation.

**Figure 1:**
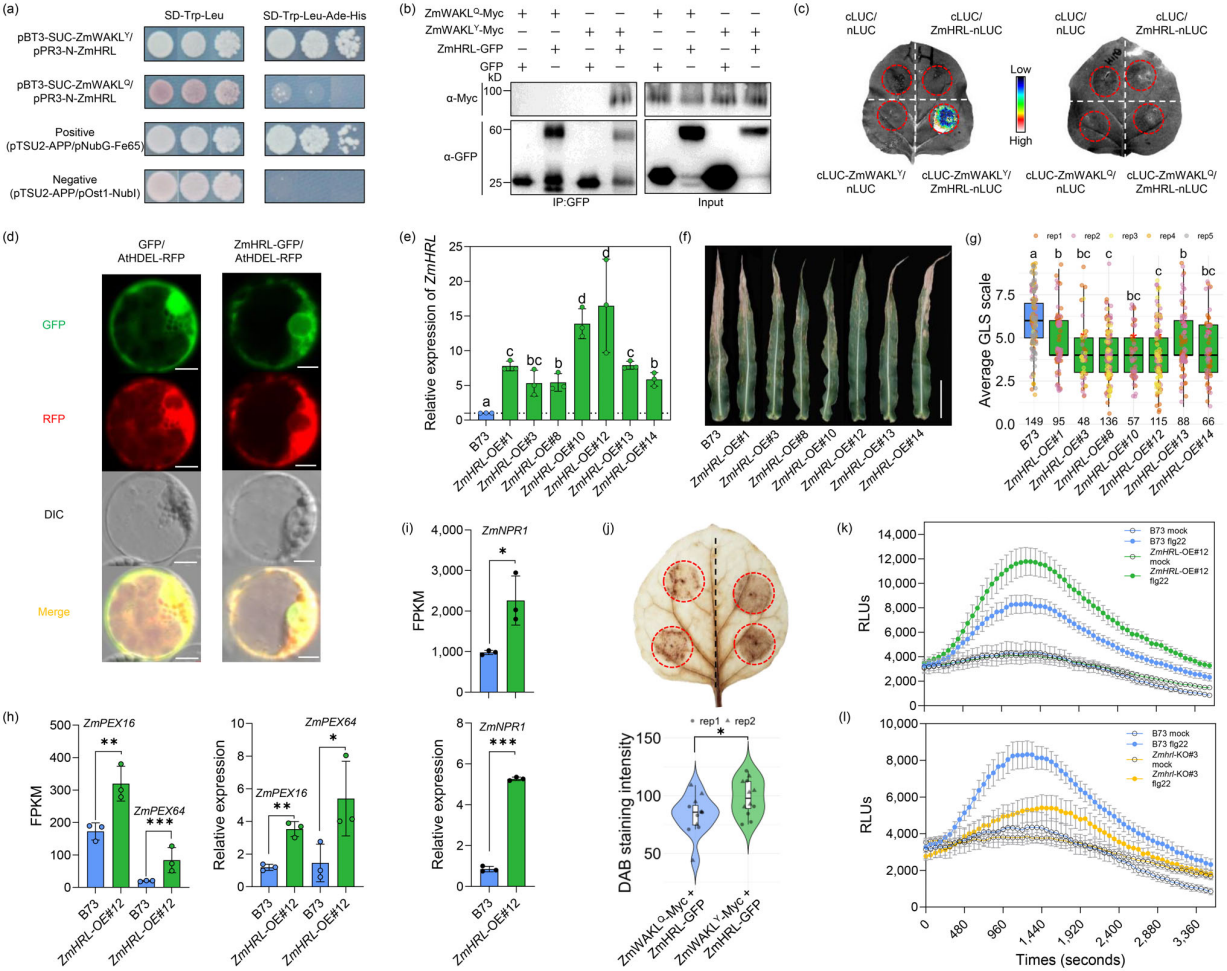
ZmHRL interacts with ZmWAKL^Y^ and positively enhances maize resistance to GLS. (a–c) ZmWAKL^Y^ interacts with ZmHRL but not with ZmWAKL^Q^ as shown by Y2H (a), Co‐IP (b), and SLC (c) assays. (d) ZmHRL‐GFP localizes to the endoplasmic reticulum (ER) in maize protoplasts. Scale bars, 10 μm. (e) Relative expression levels of *ZmHRL* in transgenic and recipient plants. (f, g) GLS symptoms (f) and disease severity scales (g) of B73 and *ZmHRL* overexpression lines. Scale bar, 15 cm. (h, i) Overexpression of *ZmHRL* upregulates peroxidase genes (h) and *ZmNPR1* (i). (j) Co‐expression of *ZmHRL‐GFP* with ZmWAKL^Y^ induces more ROS accumulation than with ZmWAKL^Q^. The upper panel shows DAB staining, and the lower panel presents the statistical analysis. (k, l) Overexpression of *ZmHRL* enhances (k), while knockout reduces (l), flg22‐triggered ROS burst.

The interaction between ZmWAKL^Y^ and ZmHRL was confirmed in *N. benthamiana* using split‐luciferase complementation (SLC) and co‐immunoprecipitation (Co‐IP) assays, whereas ZmWAKL^Q^ showed no interaction (Figure 1b,c). To further explore this interaction, ZmWAKL was divided into several segments based on its functional domains (Figure S2). SLC assays revealed that ZmHRL specifically binds to the transmembrane and intracellular regions of ZmWAKL^Y^ (ZmWAKL^Y/301–630^) (Figure S2). Among eight HR‐like lesion‐inducing domain contained proteins in maize, ZmHRL is the most closely related to AtHRL, sharing 54.5% identity and conserved structure (Figure S3). AtHRL is predicted to localize to the endoplasmic reticulum (ER) (https://www.arabidopsis.org). Consistently, ZmHRL‐GFP co‐localizes with the ER marker AtHEDL‐RFP in maize protoplasts, indicating ER membrane localization (Figure 1d).

To evaluate the role of *ZmHRL* in GLS resistance, we generated seven independent *ZmHRL*‐overexpressing (*ZmHRL*‐OE) lines in the GLS‐susceptible B73 background. These events were self‐pollinated to generate homozygous transgenic lines and crossed with the susceptible line Q11 to produce T_1_BC_1_F_1_ populations. Transcript analysis confirmed higher *ZmHRL* expression in homozygous transgenic lines (Figure 1e). These lines showed enhanced GLS resistance, with smaller lesions and lower disease severity compared with B73 (Figure 1f,g). Similarly, *ZmHRL*‐OE plants in the T_1_BC_1_F_1_ populations exhibited reduced GLS scale relative to their non‐transgenic siblings (Figure S4). To further investigate its function, we used the clustered regularly interspaced short palindromic repeats (CRISPR)/CRISPR‐associated protein 9 (Cas9) to generate three *Zmhrl* knockout mutants with 2, 5, or 13 bp deletions in the B73 background (Figure S5a). All mutants exhibited significantly reduced GLS resistance compared with B73 (Figure S5b,c). These findings indicate *ZmHRL* plays a critical role in GLS resistance in maize.

RNA‐seq analysis of *ZmHRL*‐OE#12 and B73 identified 1086 upregulated and 1124 downregulated differentially expressed genes (DEGs) (Figure S6a). Gene ontology (GO) analysis revealed significant enrichment in oxidoreductase activity and calcium ion binding (Figure S6b). Notably, all nine genes within the oxidoreductase complex (GO:1990204) were downregulated, potentially leading to peroxide accumulation (Figure S6c). Plants generate reactive oxygen species (ROS) in response to environmental stimuli by activating oxidases and peroxidases. Consistent with this, two oxidases and two peroxidases were upregulated (Figure S6c). Furthermore, 31 calcium ion‐binding genes were upregulated, including 12 encoding calcium‐dependent protein kinases (CPKs), among which *ZmCPK39* (*Zm00001d015100*) has been previously linked to multiple foliar disease resistance (Zhu *et al*., 2024) (Figure S6d). KEGG analysis showed enrichment in plant immunity‐related pathways, including plant hormone signaling, phenylpropanoid biosynthesis, MAPK signalling, and plant–pathogen interactions (Figure S6e). Finally, RT‐qPCR confirmed RNA‐seq results for two peroxidase genes and the immune marker gene *ZmNPR1* (Figure 1h,i).

Transient expression of *ZmHRL‐GFP* in *N. benthamiana* led to increased H_2_O_2_ accumulation (Figure S7a,b). Co‐expression of *ZmHRL‐GFP* with the resistant allele *ZmWAKL* ^*Y*^ resulted in higher H_2_O_2_ levels than with the susceptible allele *ZmWAKL* ^*Q*^ (Figure 1j). Luminol‐based assays showed higher flg22‐induced ROS production in the resistant NIL‐Y32 (*ZmWAKL* ^*Y*^) compared with the susceptible NIL‐Q11 (*ZmWAKL* ^*Q*^), while chitin‐induced ROS levels were comparable (Figure S7c,d). This suggests that *ZmWAKL* is associated with the response to flg22 but not chitin. Furthermore, flg22‐triggered ROS production was elevated in *ZmHRL*‐OE lines but reduced in *Zmhrl* mutants compared with their recipient line B73 (Figure 1k,l). Sequence alignment revealed that ZmWAKL^B^ (from B73) is highly similar to ZmWAKL^Y^ but contains multiple amino acid variations in both the extracellular and intracellular domains (Figure S8). These findings suggest that ZmWAKL^B^, like ZmWAKL^Y^, may participate in immune signalling via ZmHRL to trigger ROS burst, albeit with differing efficiency.

In conclusion, ZmHRL, an ER‐localized protein, interacts with ZmWAKL to regulate ROS production, thereby enhancing GLS resistance. This study highlights the critical role of ER‐localized proteins in plant defence, providing new insights for improving disease resistance in crops. Future research should elucidate how *ZmHRL* coordinates ER functions and immune responses to strengthen plant resilience.

## Conflict of interest

The authors have declared no conflict of interest.

## Author contributions

M.X. and T.Z. designed the experiments; T.Z., C.G., and M.Z. performed the experiments; T.Z., M.Z., Q.Z., S.D., and M.X. analysed the data; T.Z. and M.X. wrote the manuscript; All authors discussed the results and commented on the manuscript.

## Supporting information


**Figure S1** Screening and identification of *ZmHRL*.
**Figure S2** Searching for ZmHRL binding region in ZmWAKL^Y^ and ZmWAKL^Q^.
**Figure S3** Phylogenetic analysis, conserved domain architecture, and structural prediction of HRLs.
**Figure S4** Overexpression of *ZmHRL* enhanced GLS resistance in backcross populations.
**Figure S5** Knockout of *ZmHRL* by *CRISPR/Cas9* results in altered GLS resistance.
**Figure S6** RNA‐seq analysis between *ZmHRL*‐OE#12 and B73.
**Figure S7** *ZmHRL*‐GFP ectopic expression in *N. benthamiana* enhances H_2_O_2_ accumulation and elicitors induced ROS burst in Q11 background.
**Figure S8** Sequence alignment of ZmWAKL from B73, Y32, and Q11.
**Table S1** Candidates ZmWAKL interacting with proteins and their putative functions.
**Table S2** Primers used in this study.

**Table S3** The source data for all figures.

## Data Availability

The data that supports the findings of this study are available in the [Link], [Link] of this article.

## References

[pbi70239-bib-0001] Luan, H. , Liao, W. , Niu, H. , Cui, X. , Chen, X. and Zhi, H. (2019) Comprehensive analysis of soybean mosaic virus P3 protein interactors and hypersensitive response‐like lesion‐inducing protein function. Int. J. Mol. Sci. 20, 3388. 31295900 10.3390/ijms20143388 PMC6678280

[pbi70239-bib-0002] Meisel, B. , Korsman, J. , Kloppers, F.J. and Berger, D.K. (2009) Cercospora zeina is the causal agent of grey leaf spot disease of maize in southern Africa. Eur. J. Plant Pathol. 124, 577–583.

[pbi70239-bib-0003] Mueller, D.S. , Wise, K.A. , Sisson, A.J. , Allen, T.W. , Bergstrom, G.C. , Bissonnette, K.M. , Bradley, C.A. *et al*. (2020) Corn yield loss estimates due to diseases in the United States and Ontario, Canada, from 2016 to 2019. Plant Health Progr. 21, 238–247.

[pbi70239-bib-0004] Park, S.R. , Moon, S.J. , Shin, D.J. , Kim, M.G. , Hwang, D.J. , Bae, S.C. , Kim, J.G. *et al*. (2010) Isolation and characterization of rice *OsHRL* gene related to bacterial blight resistance. Plant Pathol. J. 26, 417–420.

[pbi70239-bib-0005] Savary, S. , Willocquet, L. , Pethybridge, S.J. , Esker, P. , McRoberts, N. and Nelson, A. (2019) The global burden of pathogens and pests on major food crops. Nat. Ecol. Evol. 3, 430–439. 30718852 10.1038/s41559-018-0793-y

[pbi70239-bib-0006] Wu, X. , Lai, Y. , Rao, S. , Lv, L. , Ji, M. , Han, K. , Weng, J. *et al*. (2021) Genome‐wide identification reveals that Nicotiana benthamiana hypersensitive response (HR)‐like lesion inducing protein 4 (NbHRLI4) mediates cell death and salicylic acid‐dependent defense responses to Turnip mosaic virus. Front. Plant Sci. 12, 627315. 34113359 10.3389/fpls.2021.627315 PMC8185164

[pbi70239-bib-0007] Yu, Y. , Shi, J. , Li, X. , Liu, J. , Geng, Q. , Shi, H. , Ke, Y. *et al*. (2018) Transcriptome analysis reveals the molecular mechanisms of the defense response to gray leaf spot disease in maize. BMC Genomics, 19, 1–7. 30305015 10.1186/s12864-018-5072-4 PMC6180411

[pbi70239-bib-0008] Zhong, T. , Zhu, M. , Zhang, Q. , Zhang, Y. , Deng, S. , Guo, C. , Xu, L. *et al*. (2024) The ZmWAKL–ZmWIK–ZmBLK1–ZmRBOH4 module provides quantitative resistance to gray leaf spot in maize. Nat. Genet. 56, 315–326. 38238629 10.1038/s41588-023-01644-z PMC10864183

[pbi70239-bib-0009] Zhu, M. , Zhong, T. , Xu, L. , Guo, C. , Zhang, X. , Liu, Y. , Zhang, Y. *et al*. (2024) The ZmCPK39–ZmDi19–ZmPR10 immune module regulates quantitative resistance to multiple foliar diseases in maize. Nat. Genet. 56, 2815–2826. 39496881 10.1038/s41588-024-01968-4 PMC11631770

