# Supplementary material for: A hypersensitive response‐like lesion‐inducing protein modulates basal immunity against grey leaf spot in maize
Source: Plant Biotechnol J. 2025 Jul 21;23(11):4732–4. doi: 10.1111/pbi.70239 (PMC12576461; doi:10.1111/pbi.70239)
Supplement: Supplementary file 1 — Figure S1 Screening and identification of ZmHRL. Figure S2 Searching for ZmHRL binding region in ZmWAKLY and ZmWAKLQ. Figure S3 Phylogenetic analysis, conserved domain architecture, and structural prediction of HRLs. Figure S4 Overexpression of ZmHRL enhanced GLS resistance in backcross populations. Figure S5 Knockout of ZmHRL by CRISPR/Cas9 results in altered GLS resistance. Figure S6 RNA‐seq analysis between ZmHRL‐OE#12 and B73. Figure S7 ZmHRL‐GFP ectopic expression in N. benthamiana enhances H2O2 accumulation and elicitors induced ROS burst in Q11 background. Figure S8 Sequence alignment of ZmWAKL from B73, Y32, and Q11. Table S1 Candidates ZmWAKL interacting with proteins and their putative functions. Table S2 Primers used in this study. [file PBI-23-4732-s001.docx]

**Supplemental information includes Materials and Methods, 7 figures and 2 tables.**

**Materials and Methods**

**Plant materials and field assay**

In our previous study, we cloned a major quantitative disease-resistant gene, *ZmWAKL*, from a segregating population derived from a cross between the highly GLS-resistant inbred line Y32 and GLS-susceptible inbred line Q11 (Zhang et al., 2012; Zhong et al., 2024). The susceptible parent Q11 was used as the recurrent parent in this study. Field experiments were conducted in Beijing. Transgenic plants were grown in rows, with each row measuring 4 meters in length with 0.5 meters between rows and 0.25 meters between plants within a row. All plants were artificially inoculated with the pathogenic fungus *C. zeina* to induce gray leaf spot (GLS). The fungal inoculum was prepared by culturing *C. zeina* on potato dextrose agar (PDA) medium. Spores were washed off with ddH_2_O to prepare a spore inoculum at a concentration of 5×10^3^ spores/ml. At the ten-leaf stage, plants were inoculated by pouring 5-ml inoculum into the leaf whorl. For gene expression analysis, one leaf punch was collected from the middle of the inoculated leaf of each plant. Samples for each genotype were composed of three pooled leaf punches, which were immediately stored at −80 °C.

GLS symptoms are scored two times at one-week intervals, starting 2 weeks after pollination. The GLS severity was assessed by the number and size of lesions on the leaves of the entire plant. GLS scale was rated using the following scale: 1 (highly resistant), 3 (resistant), 5 (intermediate resistant/susceptible), 7 (susceptible), and 9 (highly susceptible). Due to the inconsistency in scoring timing and disease severity across different field tests, the results for B73 varied between different plots within the same field. Therefore, comparisons between the transgenic lines and B73 were conducted within the same field region. To minimize regional and subjective variation, we used the average of the two scores as the final phenotypic value for subsequent analysis.

For transgenic functional validation in the Q11 background, we crossed T_1_ transgenic events with Q11, followed by backcrossing to Q11 to generate the T_1_BC_1_F_1_ population. In this segregating population, the endogenous *ZmHRL* locus exhibited either a homozygous *ZmHRL^Q11^*/*ZmHRL^Q11^* or heterozygous *ZmHRL^Q11^*/*ZmHRL^B73^* genotype. Meanwhile, the transgene was segregating, and plants were either transgene-positive (Transgenic) or transgene-negative (Non-transgenic). For analysis, we grouped plants based on transgene presence. Importantly, both the transgenic and non-transgenic groups contained comparable proportions of the two endogenous *ZmHRL* genotypes, effectively controlling for potential effects from the endogenous *ZmHRL*.

**Yeast two-hybrid (Y2H) assay**

To identify ZmWAKL^Y^-interacting proteins, the full-length coding sequence (CDS) of *ZmWAKL^Y^* without the signal peptide was cloned into the pBT3-SUC vector to create a bait construct. This bait construct was used to screen a Y32 cDNA library using the split-ubiquitin yeast two-hybrid (Y2H) technique, following the manufacturer’s user guide (Clontech). The yeast strain NMY51 was used in this assay. The full-length CDS of the potential interaction proteins were cloned into the pPR3-N vector to serve as prey. The bait and prey vectors were co-transformed into the NMY51 strain, and protein-protein interactions were tested on synthetic defined (SD) medium lacking the appropriate nutrients.

**Split-luciferase complementation (SLC) assay**

The full-length CDS of *ZmHRL* and two *ZmWAKL* alleles, along with their respective fragments, were cloned into the JW771-35S-NLuc and JW772-35S-CLuc vectors to generate the *ZmHRL-nLuc* and *cLuc-ZmWAKL* fusion constructs. These constructs were individually transformed into *Agrobacterium* strain EHA105. Bacterial cultures carrying the appropriate constructs were collected by centrifugation at 1,200 g for 15 min at room temperature and resuspended in an infiltration buffer (10 mM MES, pH 5.6, 10 mM MgCl_2_, 150 μM acetosyringone). Equal amounts of *Agrobacterium* cultures for each cLUC and nLUC construct were mixed and incubated for 3 h at 28ºC. The mixture was infiltrated into three- to four-week-old *N. benthamiana* leaves. At 2.5 days post-infiltration, the plants were treated with 1 mM beetle luciferase (Beetle luciferin, Promega). Luminescence signals were measured using a Chemiluminescent Imaging System (Tanon-5200).

**Co-immunoprecipitation (Co-IP) assay**

A Co-IP assay was carried out to verify protein-protein interactions in planta. The full-length CDS of *ZmHRL* and two *ZmWAKL* alleles were amplified with gene-specific primers and cloned into the *pSuper1300-Myc* or *pSuper1300-GFP* vectors, respectively. The resulting constructs were transformed into *Agrobacterium* strain EHA105 and transiently co-infiltrated in *N. benthamiana* leaves. The leaf tissues were sampled three days post-infiltration, ground into powder in liquid nitrogen, resuspended in membrane protein extraction buffer (50 mM Tris-HCl, pH 7.5, 150 mM NaCl, 0.2% [v/v] Triton X-100, 5 mM DTT, 1 mM PMSF, and 1% [w/v] protease inhibitor cocktail), and incubated on ice for 30 min. The suspension was then centrifuged at 13,000 g for 20 min at 4 ºC. The Supernatant was incubated with anti-GFP magnetic agarose beads for 2 h at 4ºC. The beads were washed five times for 5 min each in extraction buffer. Proteins were eluted by boiling the beads in 60 μL extraction buffer containing 1× SDS-PAGE sample buffer for 5 min at 99ºC. The Myc-tagged and GFP-tagged proteins were detected using immunoblotting with anti-Myc (Abclonal, AE010) and anti-GFP (Abclonal, AE012) antibody, respectively.

**Phylogenetic analysis of HRLs**

Protein sequences containing HR-like lesion-inducing domains were downloaded from the Gramene database (https://www.gramene.org/). Additionally, HRLs protein sequences from other species were obtained from the NCBI database (<https://www.ncbi.nlm.nih.gov/>). A phylogenetic tree of HRLs was constructed using the maximum-likelihood method with default parameters in MEGA 7.0 (<http://www.megasoftware.net>). Bootstrap values from 1000 pseudo-replicates were used to provide support for the nodes in the phylogenetic tree. The conserved domains were predicted using the NCBI Conserved Domains Database (<https://www.ncbi.nlm.nih.gov/Structure/cdd/wrpsb.cgi>). Protein structures were predicted using AlphaFold (https://alphafoldserver.com/).

**Subcellular localization**

The CDS of *ZmHRL* was inserted into *pSuper1300-GFP* to generate the fusion construct. The *ZmHRL-GFP* construct was transformed into maize protoplasts via polyethylene glycol (PEG)-mediated transfection. Maize protoplasts were isolated from B73 leaf tissues and transfected with the *ZmHRL-GFP* construct according to a previously described protocol (Yoo et al., 2007). The GFP fluorescence was visualized under a confocal microscope (Zeiss).

**Genetic transformation**

To construct overexpression vector, the *ZmHRL* coding sequence (CDS) was cloned into the expression vector *pBCXUN-Myc* driven by the maize ubiquitin promoter as previously described (Chen et al., 2022). To knock out the endogenous *ZmHRL* gene, CRISPR/Cas9 vectors were constructed following the protocol previously described (Xing et al., 2014). These vectors were transferred into *Agrobacterium* strain EHA105 and then transformed into the maize inbred line B73. The genotypes were confirmed through PCR or sequencing of standard PCR products. All T_1_ positive or edited plants from each transgenic event were self-pollinated to produce homozygous transgenic plants or backcrossed to the susceptible parent Q11 to produce backcross populations for functional verification.

**RNA-seq analysis**

Leaves from *ZmHRL*-OE#12 and B73 plants were used for RNA sequencing. For each genotype, three biological replicates were included, each consisting of five independent plants. Total RNA extraction and poly(A) RNA isolation were performed according to the manufacturer's protocol (Invitrogen). For RNA‐seq library construction, 100 ng of poly(A) messenger RNA was fragmented and processed as recommended by the manufacturer (TruSeq RNA Library Prep Kit v2, Illumina) and sequenced on the Illumina Hiseq3000. The clean reads were then mapped to the masked maize genome in the Ensemble *Zea_mays*, AGPv3.26 database (http://plants.ensembl.org/Zea_mays/Info/Index). Gene expression levels were calculated and normalized based on FPKM (fragments per kilobase of exon model per million mapped reads) using Cufflinks software, version 2.1.1.

Differential expression analysis between the two groups (ZmHRL-OE#12 and B73), each with three biological replicates, was performed using the DESeq2 R package (1.20.0). DESeq2 employs statistical models based on the negative binomial distribution to identify differentially expressed genes (DEGs) in digital gene expression data. The resultant *P*-values were adjusted using the Benjamini and Hochberg’s method for controlling the false discovery rate. Genes with an adjusted *P*-value <0.05 were assigned as differentially expressed genes (DEGs). Gene Ontology (GO) enrichment analysis of DEGs was implemented by the clusterProfiler R package, in which gene length bias was corrected. GO terms with a corrected *P*-value < 0.05 were considered significantly enriched by DEGs. The clusterProfiler R package was also used to statistically test the enrichment of DEGs in KEGG pathways.

**Histochemical staining of H_2_O_2_**

The histochemical analysis of H_2_O_2_ accumulation was performed using 3,3′-diaminobenzidine (DAB) staining as described previously (Daudi & O'Brien, 2012). The leaf samples were vacuum-infiltrated with DAB staining buffer (1 mg/ml) for 2 h and then incubated at 30°C in darkness for 12–16 h. The stained leaves were stored in 10% glycerol prior to observation.

**ROS burst detection**

Reactive oxygen species (ROS) production was measured using a luminol-based assay as described previously with minor modifications (Wang et al., 2021). Leaf discs were collected from three-leaf stage plants using a 3-mm diameter punch and incubated in 100 ul 1% DMSO in a 96-well plate overnight. At least eight plants per line were sampled. Next, 1% DMSO was replaced by 2× L-012 in 0.05% Silwet L-77 (Wako Pure Chemical Industries Ltd, Osaka, Japan; 15 mg L-012 in 1 ml 200 mM KOH is 100× L-012). Then 2× HRP solution (Sigma; 25 mg HRP in 2.5 ml is 100× HRP) containing either 2.5 mM flg22 or 50 mg/ml chitin was added to initiate the reaction. The luminescence was recorded every 30 or 60 s for at least 1 h using a GLOMAX96 Luminometer (Promega).

**References**

Chen, W., Chen, L., Zhang, X., Yang, N., Guo, J., Wang, M., Ji, S. *et al*. (2022) Convergent selection of a WD40 protein that enhances grain yield in maize and rice. *Science* **375**, eabg7985.

Daudi, A., O'Brien, J.A. (2012) Detection of Hydrogen Peroxide by DAB Staining in *Arabidopsis* Leaves. *Bio. Protoc*. **2**(18):e263.

Wang, H., Hou, J., Ye, P., Hu, L., Huang, J., Dai, Z., Zhang, B. *et al*. (2021) A teosinte-derived allele of a MYB transcription repressor confers multiple disease resistance in maize. *Mol. Plant*, **14**(11), 1846-1863.

Xing, H.L., Dong, L., Wang, Z.P., Zhang, H.Y., Han, C.Y., Liu, B., Wang, X.C. *et al*. (2014) A CRISPR/Cas9 toolkit for multiplex genome editing in plants. *BMC Plant Biol*. **14**, 327.

Yoo, S.D., Cho, Y.H., Sheen, J. (2007) *Arabidopsis* mesophyll protoplasts: a versatile cell system for transient gene expression analysis. *Nat. protoc*. **2**, 1565-1572.

Zhang, Y., Xu, L., Fan, X., Tan, J., Chen, W., Xu, M. (2012) QTL mapping of resistance to gray leaf spot in maize. *Theor. Appl. Genet.* **125**,1797-1808.

Zhong, T., Zhu, M., Zhang, Q., Zhang, Y., Deng, S., Guo, C., Xu, L. *et al*. (2024) The ZmWAKL–ZmWIK–ZmBLK1–ZmRBOH4 module provides quantitative resistance to gray leaf spot in maize. *Nat. Genet.* **56**, 315–326.

**Legends of supplemental figures**

**
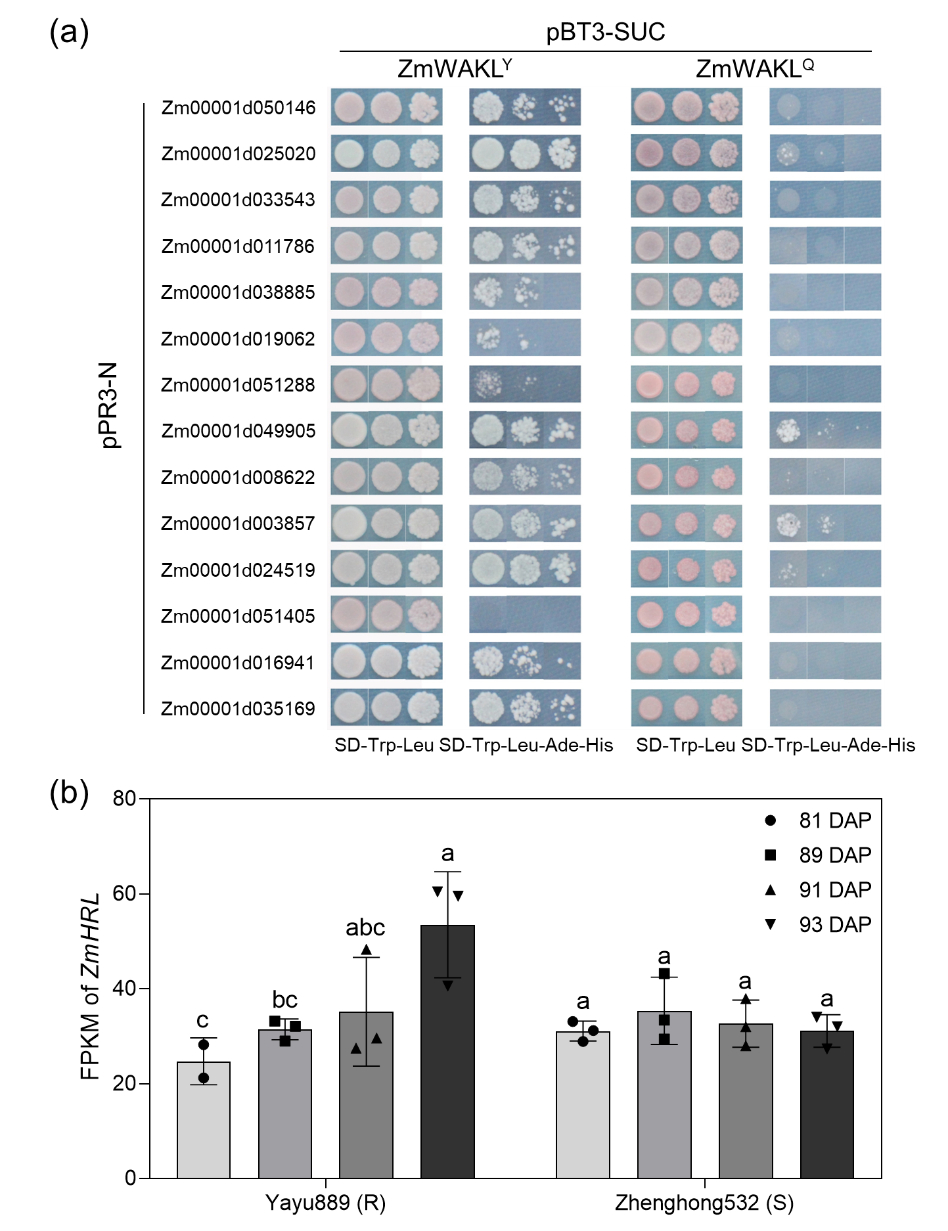
**

**Figure S1 Screening and identification of *ZmHRL*.**

(a) The interaction of ZmWAKL^Y^ and ZmWAKL^Q^ with potential interactors were validated by using the split-ubiquitin-based membrane Y2H system. SD: synthetic defined medium. The yeast strain NMY51 was used here.

(b*) ZmHRL* expression was induced by *C. zeina* in a resistant background. Yayu889, resistant cultivar; Zhenghong532, susceptible cultivar. DAP: Days after planting. The raw data were downloaded from <https://plantrnadb.com>. Different lowercase letters indicate significant differences (*P* < 0.05) based on one-way ANOVA.

**
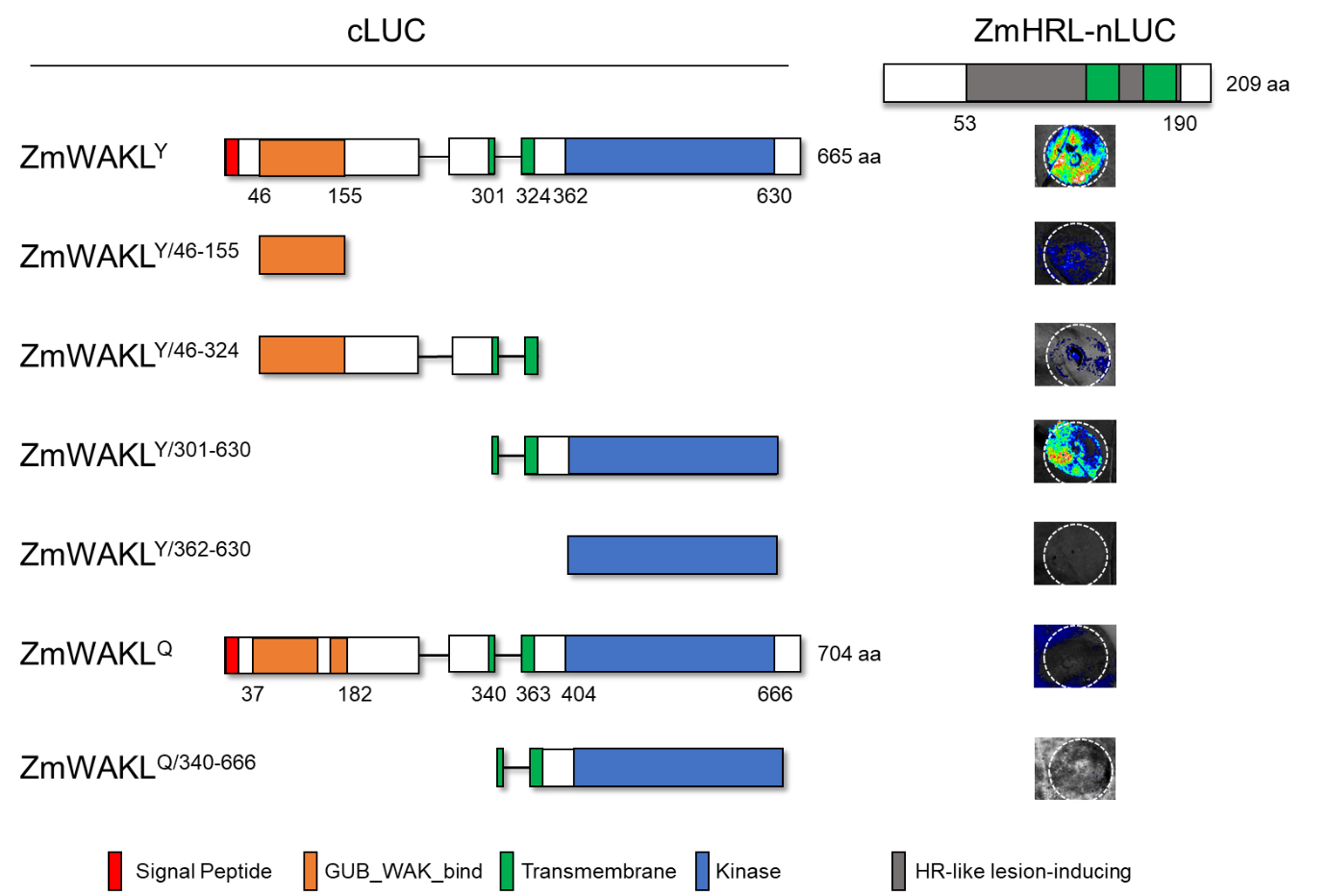
**

**Figure S2 Searching for the ZmHRL binding region in ZmWAKL^Y^ and ZmWAKL^Q^.**

To reveal the ZmHRL binding regions, ZmWAKL was divided into multiple segments based on its conserved functional domains. These domains were predicted using the NCBI Conserved Domains Database (<https://www.ncbi.nlm.nih.gov/Structure/cdd/wrpsb.cgi>). Images on the right show the SLC assays where various cLUC-tagged ZmWAKL or ZmWAKL gene segments were co-expressed with the ZmHRL-nLUC in *N. benthamiana* leaves. The transmembrane and intracellular domains of ZmWAKL^Y^ could interact with ZmHRL, whereas the corresponding domains of ZmWAKL^Q^ did not.

**
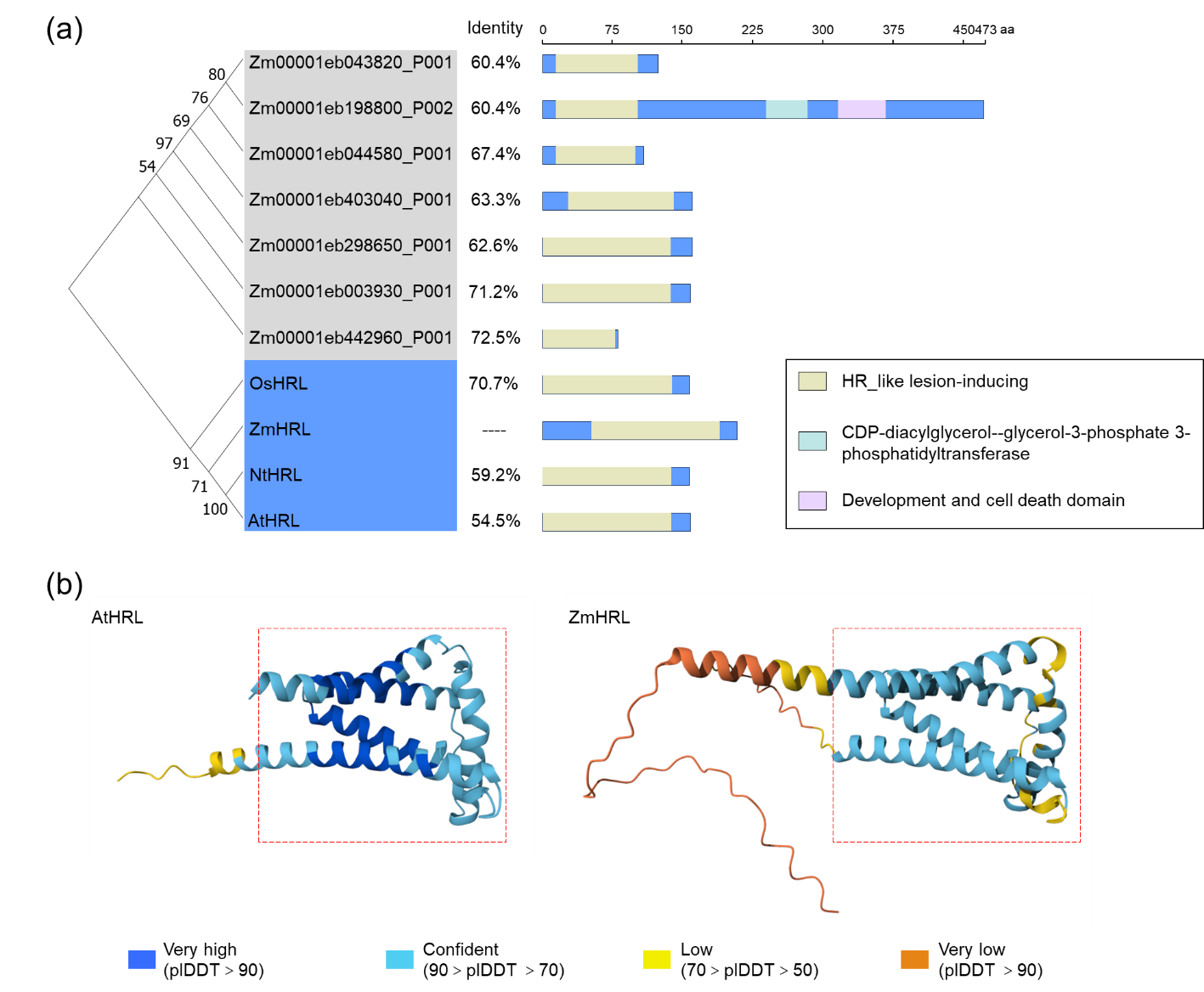
**

**Figure S3** **Phylogenetic analysis, conserved domain architecture, and structural prediction of HRLs .**

(a) Phylogenetic analysis and conserved domain architecture of HRLs across plant species. This study analyzed maize HR-like lesion-inducing domains proteins, as well as HRL proteins from other species reported in the literature, including AtHRL, NtHRL and OsHRL. All maize HR-like lesion-inducing domain-containing protein sequences were retrieved from the Gramene database (https://www.gramene.org/). The HRL protein sequences of other species were obtained from the NCBI database using their GenBank accessions (<https://www.ncbi.nlm.nih.gov/>). AtHRL. CAB10221; NtHRL, AAC49975; OsHRL, FJ548850. The phylogenetic tree of HRLs was constructed using the maximum-likelihood method with default parameters in MEGA 7.0 (http://www.megasoftware.net). Bootstrap values from 1000 pseudo-replicates were used to provide support for the nodes in the phylogenetic tree. The conserved domains were predicted using the NCBI Conserved Domains Database (<https://www.ncbi.nlm.nih.gov/Structure/cdd/wrpsb.cgi>). The identity values represent the sequence identity between ZmHRL and other HRLs. The full-length protein sequences of ZmHRL and AtHRL share 54.5% identity (85/156). For the HR_like lesion inducing domain, the identity is 56.5% (78/138). Os, *Oryza sativa*; At, *Arabidopsis thaliana*; Zm*, Zea mays*; Nt, *Nicotiana tabacum*.

(b) AlphaFold-based structural prediction of AtHRL and ZmHRL. Red dashed boxes highlight regions of structural similarity between the two proteins. Protein structures were predicted using AlphaFold (<https://alphafoldserver.com/>). The plDDT (predicted local distance difference test) is a per-residue measure of local confidence.

**
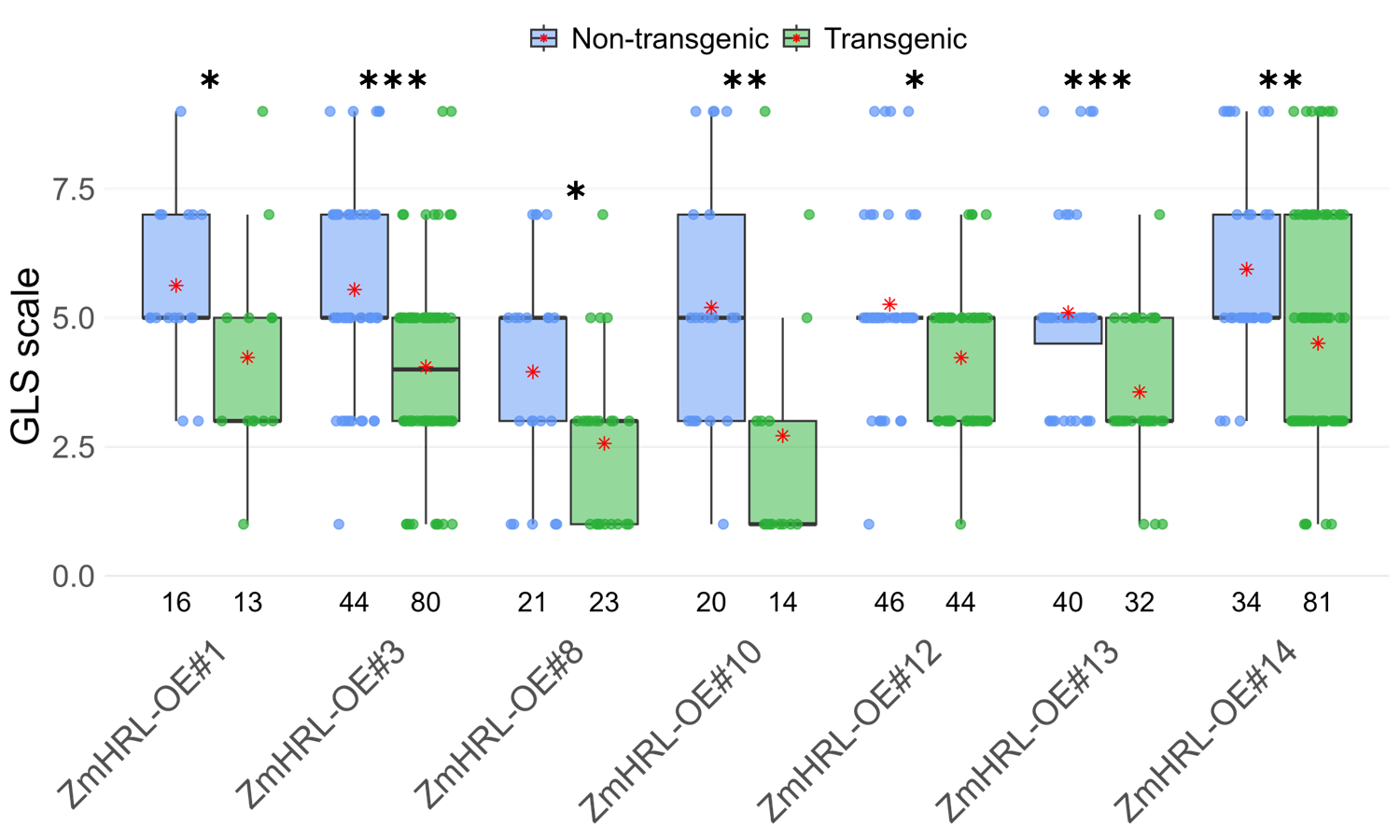
**

**Figure S4 Overexpression of *ZmHRL* enhances GLS resistance in backcross populations.**

GLS scales of transgenic and non-transgenic plants in the T_1_BC_1_F_1_ backcross population. Data are displayed as box and whisker plots with individual data points. The red asterisk denotes the mean, while the box limits indicate the interquartile range. Statistical significance was determined by a two-sided Student’s *t*-test.

**
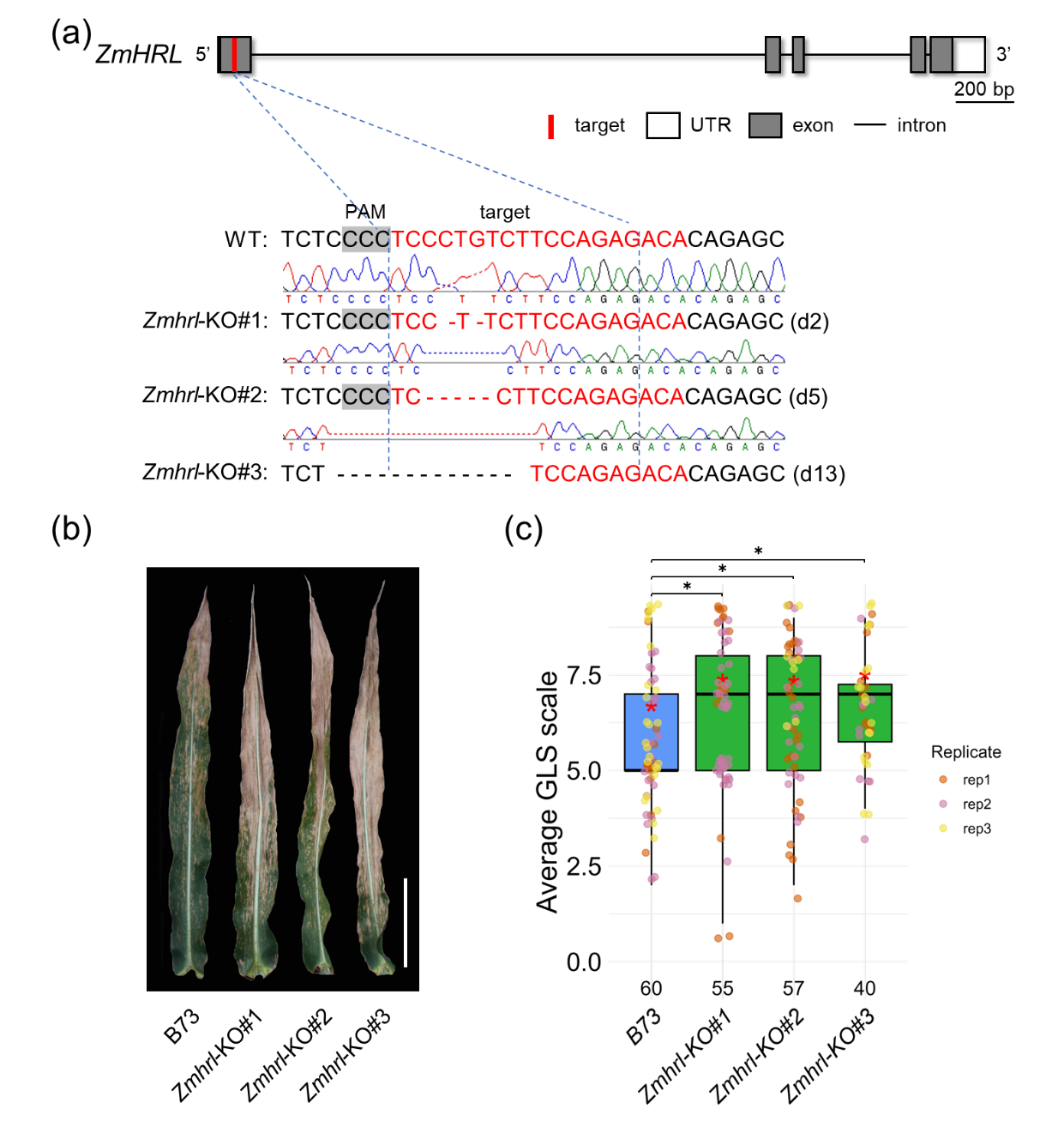
**

**Figure S5 Knockout of *ZmHRL* by *CRISPR/Cas9* results in altered GLS resistance.**

(a) Schematic diagram of *ZmHRL* and the sgRNA target site in the first exon for *CRISPR/Cas9*-mediated editing. The sequences of three homozygous knockout lines with deletion (KO#1,2,3) are shown. The wild-type B73 sequence is displayed at the top. The target sites and PAM sequences are highlighted in red and gray, respectively, with deletions indicated by dashes.

(b,c) GLS symptoms (b) and scale (c) of B73 and three homozygous knockout lines. The knockout lines exhibit significantly reduced GLS resistance. Scale bar, 15 cm.

Data in (c) are displayed as box and whisker plots with individual data points. Each dot color corresponds to a different biological replicate. The asterisk denotes the mean, while the box limits indicate the interquartile range. The center line indicates the median, and statistical significance was determined by a two-sided Student’s *t*-test.

**
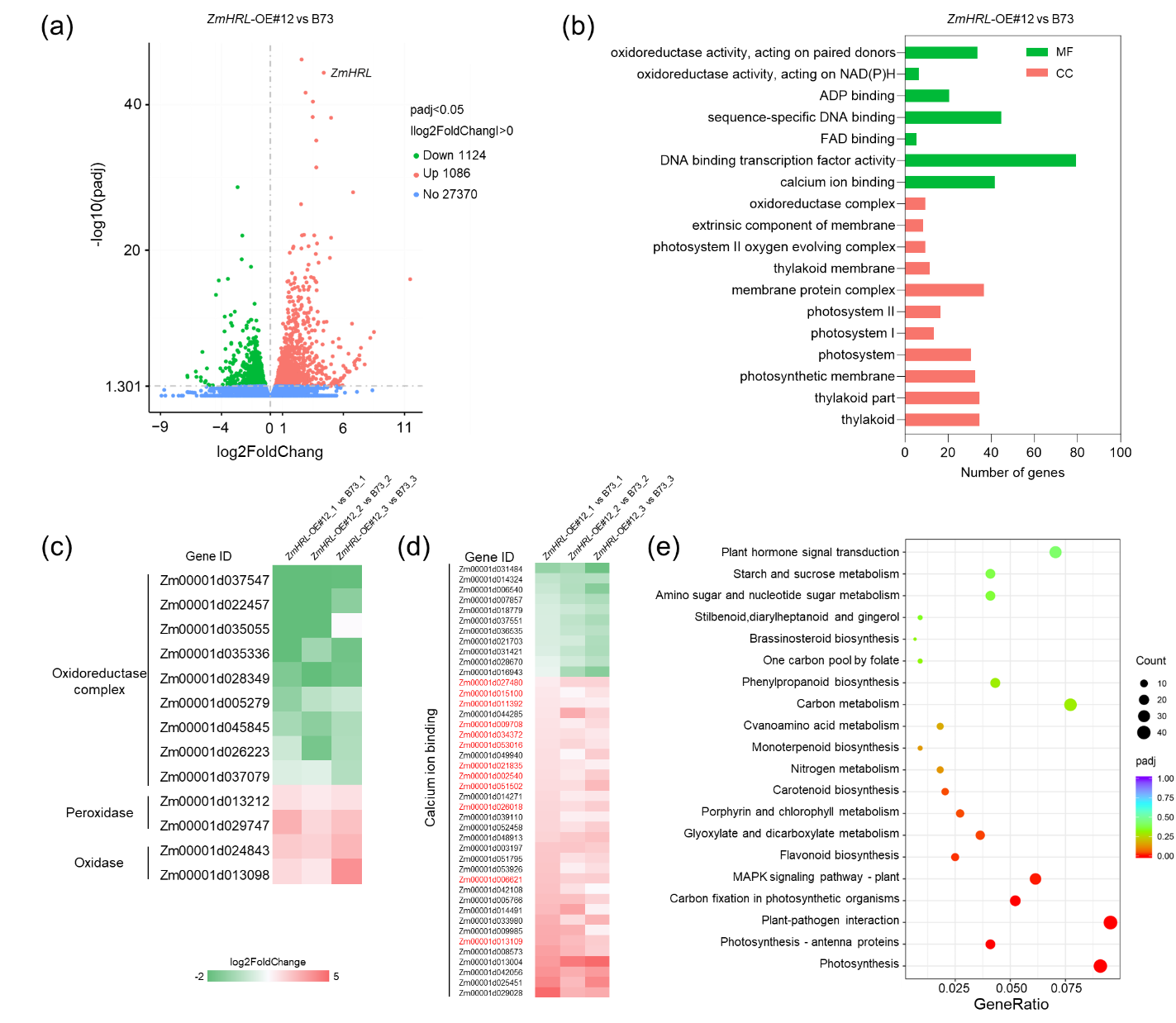
**

**Figure S6 RNA-seq analysis between *ZmHRL*-OE#12 and B73.**

(a) Volcano plots showing differentially expressed genes (DEGs) between *ZmHRL*-OE#12 and B73. DEGs were identified using a threshold of padj < 0.05 and an absolute log2-fold change > 0. Green dots represent genes that were significantly down-regulated; red dots represent genes that were significantly up-regulated; blue dots represent genes with no significant differences in expression.

(b) GO analysis of DEGs between *ZmHRL*-OE#12 and B73. MF, molecular function; CC, cellular component. Both CC and MF categories show enrichment of genes in oxidoreductase activity-related pathways.

(c) Heatmap illustrates 13 DEGs involved in the oxidoreductase complex and oxidase activity. The changes in gene expression are represented as log2FoldChange values. Red indicates up-regulation, while green indicates down-regulation.

(d) Heatmap depicting DEGs enriched in calcium ion binding genes. Red fonts indicate calcium-dependent protein kinases, all of which are up-regulated. The changes in gene expression are represented as log2FoldChange values. Red indicates up-regulation, while green indicates down-regulation.

(e) KEGG pathway enrichment analysis of DEGs between *ZmHRL*-OE#12 and B73. The size of each circle indicates the number of enriched genes, while different colors indicate the padj value.


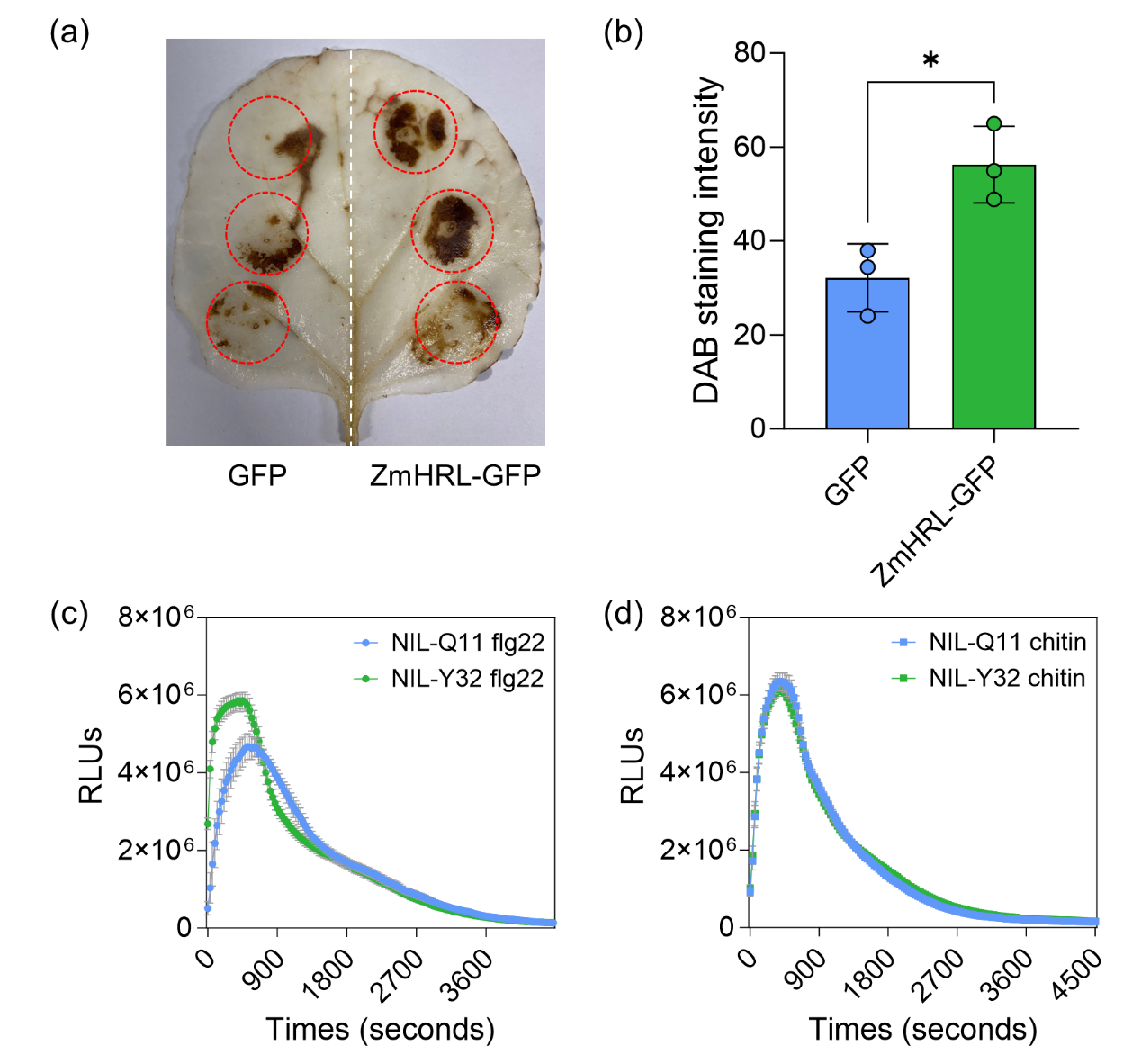


**Figure S7 *ZmHRL*-GFP expression-driven H_2_O_2_ accumulation in *N. benthamiana* and elicitor-induced ROS burst in the Q11 background.** (a) DAB staining shows H_2_O_2_ accumulation in *N. benthamiana* leaves. The GFP empty vector serves as a control.

(b) Quantification of DAB staining intensity. Statistical significance was determined by a two-sided Student’s *t*-test.

(c,d) Elicitor-induced reactive oxygen species (ROS) production in the third leaves of NIL-Q11 and NIL-Y32. ROS production was measured as relative light units (RLU) using a luminol-based assay. Comparison of flg22- (c) and chitin-triggered (d) ROS production in the same two NILs, values are mean ± se.

**
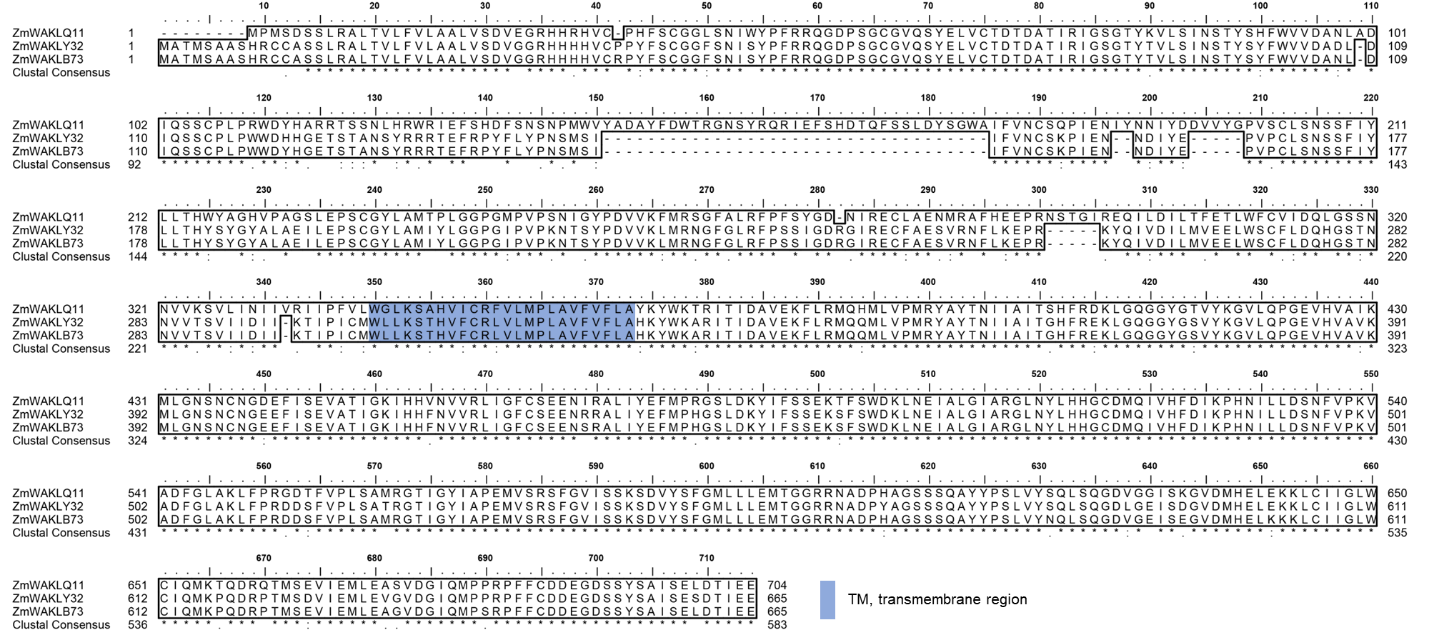
**

**Figure S8 Sequence alignment of ZmWAKL from B73, Y32, and Q11.**

Amino acid sequence alignment of ZmWAKL from B73, Y32, and Q11. Alignment was performed using MUSCLE (https://www.ebi.ac.uk/jdispatcher/msa/muscle?stype=protein).

**Table S1** **Candidate ZmWAKL-interacting proteins and their putative functions.**

| Interactor (Gene ID) | Frequency^a^ | Protein function | Transmembrane domain^b^ |
| --- | --- | --- | --- |
| Zm00001d050146 | 3 | Syntaxin-51 | Y |
| Zm00001d025020 | 4 | HR-like lesion inducing protein | Y |
| Zm00001d033543 | 2 | Integral membrane HPP family protein | Y |
| Zm00001d011786 | 2 | Cytochrome B5 isoform D | Y |
| Zm00001d038885 | 1 | Programmed cell death protein 5 | N |
| Zm00001d019062 | 1 | membrane H(+)-ATPase3 | Y |
| Zm00001d051288 | 4 | Vesicle-associated protein 1-1 | Y |
| Zm00001d049905 | 4 | ABC transporter F family member 1 | Y |
| Zm00001d008622 | 2 | Synaptic glycoprotein SC2 | Y |
| Zm00001d003857 | 1 | calnexin homolog1 | Y |
| Zm00001d024519 | 6 | rubredoxin family protein | Y |
| Zm00001d051405 | 2 | transmembrane protein | Y |
| Zm00001d016941 | 1 | RING/FYVE/PHD zinc finger superfamily protein | Y |
| Zm00001d035169 | 2 | Chloroplast J-like domain 1 | Y |

**^a^** A total of 80 clones was selected for sequencing.

^b^ ‘Y’ indicates that the protein contains transmembrane domain, and ‘N’ means that the protein does not have transmembrane domain. Transmembrane domains were predicted by DeepTMHMM (https://dtu.biolib.com/DeepTMHMM).

**Table S2 Primers used in this study.**

|  | name | sequence (5'-3') | anotation |
| --- | --- | --- | --- |
| primers used in yeast two hybrid assay | pBT3-SUC-Y | GGCCATTACGGCC CGACATCATCATCATGTTTG | the N-terminal does not contain signal peptide |
|  |  | GGCCGAGGCGGCCCC CTCTTCTATTGTATCCGATT |  |
|  | pBT3-SUC-Q | GGCCATTACGGCC CGACATCATCGTCATGTCTG |  |
|  |  | GGCCGAGGCGGCCCC CTCTTCTATTGTATCCAATT |  |
|  | pPR3-N-HRL | CAACGCAGAGTGGCCATTACC ATGGAAGACCGACTCGACCA | Zm00001d025020 |
|  |  | CGAATTCTCGAGAGGCCGAGG TCAGTTTGTCTTGGTTTTTG |  |
|  | pPR3-N-Zm00001d050146 | CAACGCAGAGTGGCCATTACC ATGGCATCATCTTCGGACCC | Zm00001d050146 |
|  |  | CGAATTCTCGAGAGGCCGAGG TTACAGATCAAGAGCAGTTA |  |
|  | pPR3-N-Zm00001d033543 | CAACGCAGAGTGGCCATTACC ATGGCTTTGCTGCAGAGCCA | Zm00001d033543 |
|  |  | CGAATTCTCGAGAGGCCGAGG TTAAAATGTATGTTGACCAT |  |
|  | pPR3-N-Zm00001d011786 | CAACGCAGAGTGGCCATTACC ATGTCGGGATCCAAGGTGTA | Zm00001d011786 |
|  |  | CGAATTCTCGAGAGGCCGAGG CTAGGCAGATTCCGACTTGG |  |
|  | pPR3-N-Zm00001d038885 | CAACGCAGAGTGGCCATTACC ATGGAGAACGGCGAGGAGAC | Zm00001d038885 |
|  |  | CGAATTCTCGAGAGGCCGAGG TCATCTCTTGAGGTTGAGCA |  |
|  | pPR3-N-Zm00001d019062 | CAACGCAGAGTGGCCATTACC ATGGAGGACAAGGCGTCCAA | Zm00001d019062 |
|  |  | CGAATTCTCGAGAGGCCGAGG TCACACGGTGTAGGACTGCT |  |
|  | pPR3-N-Zm00001d051288 | CAACGCAGAGTGGCCATTACC ATGAGCACCGAGTCCGGGGA | Zm00001d051288 |
|  |  | CGAATTCTCGAGAGGCCGAGG TCATCTCTTCATGAGGAACC |  |
|  | pPR3-N-Zm00001d049905 | CAACGCAGAGTGGCCATTACC ATGGTGTCGGACGCCAGCAA | Zm00001d049905 |
|  |  | CGAATTCTCGAGAGGCCGAGG TTAATCCGATAGACAAGCCT |  |
|  | pPR3-N-Zm00001d008622 | CAACGCAGAGTGGCCATTACC ATGCTGCTGCTGCTCAGAGT | Zm00001d008622 |
|  |  | CGAATTCTCGAGAGGCCGAGG TCACAGGAACGGAGGGAGAA |  |
|  | pPR3-N-Zm00001d003857 | CAACGCAGAGTGGCCATTACC ATGGGAGGGCGCGCGCTCCT | Zm00001d003857 |
|  |  | CGAATTCTCGAGAGGCCGAGG CTATGTCTCCCTTCGGGTCC |  |
|  | pPR3-N-Zm00001d024519 | CAACGCAGAGTGGCCATTACC ATGTCAGCTCGTGCCAACAG | Zm00001d024519 |
|  |  | CGAATTCTCGAGAGGCCGAGG CTACTGCAGGCCGTACACGA |  |
|  | pPR3-N-Zm00001d051405 | CAACGCAGAGTGGCCATTACC ATGGCGAAGCAGGGGGCGAA | Zm00001d051405 |
|  |  | CGAATTCTCGAGAGGCCGAGG TCAGCGAGTCCTAGTTTTGA |  |
|  | pPR3-N-Zm00001d016941 | CAACGCAGAGTGGCCATTACC ATGGGGGATCATGTCGCGGT | Zm00001d016941 |
|  |  | CGAATTCTCGAGAGGCCGAGG CTATTGGTTTAGCTCGGGGT |  |
|  | pPR3-N-Zm00001d035169 | CAACGCAGAGTGGCCATTACC ATGGCCTCCGCGACGGCGAC | Zm00001d035169 |
|  |  | CGAATTCTCGAGAGGCCGAGG TCATCGGTAGTATGAGGCTG |  |
| primers used in Co-IP assay | WAKL^Y^MYC | TCTGCAGGGGCCCGG **GTCGAC** ATGGCGACGATGTCTGCAGC |  |
|  |  | GAGCTTTTGCTCCAT **GGTACC** CTCTTCTATTGTATCCGATT |  |
|  | WAKL^Q^MYC | TCTGCAGGGGCCCGG **GTCGAC** ATGCCGATGTCTGATTCTTC |  |
|  |  | GAGCTTTTGCTCCAT **GGTACC** CTCTTCTATTGTATCCAATT |  |
|  | HRL-GFP | TCTGCAGGGGCCCGG **GTCGAC** ATGGAAGACCGACTCGACCA |  |
|  |  | GCCCTTGCTCACCAT **GGTACC** GTTTGTCTTGGTTTTTGTTG |  |
| primers used for split-luciferase complementation assay | ZmHRL-Nluc | ACGAGCTCGGTACCCG **GGATCC** TGGAAGACCGACTCGACCA |  |
|  |  | ACGCGTACGAGATCTG **GTCGAC** GTTTGTCTTGGTTTTTGTTG |  |
|  | cLUC-ZmWAKL^Y^ | TACGCGTCCCGGGGC **GGTACC** ATGGCGACGATGTCTGCAGC |  |
|  |  | CCTTGTAGTCCATTTGTT **GGATCC** TACTCTTCTATTGTATCCG |  |
|  | cLUC-ZmWAKL^Q^ | TACGCGTCCCGGGGC **GGTACC** ATGCCGATGTCTGATTCTTC |  |
|  |  | CCTTGTAGTCCATTTGTT **GGATCC** TACTCTTCTATTGTATCCA |  |
|  | cLUC-ZmWAKL^Y/46-155^ | TACGCGTCCCGGGGC **GGTACC** TCCTGCGGTGGTTTTAGCAA |  |
|  |  | CCTTGTAGTCCATTTGTT **GGATCC** CTA CGAGCAATTCACAAAGATAA |  |
|  | cLUC-ZmWAKL^Y/46-324^ | TACGCGTCCCGGGGC **GGTACC** TCCTGCGGTGGTTTTAGCAA |  |
|  |  | CCTTGTAGTCCATTTGTT **GGATCC** CTA GCTAGGAAGACAAATACTG |  |
|  | cLUC-ZmWAKL^Y/301-630^ | TACGCGTCCCGGGGC **GGTACC** TGGCTTCTGAAATCTACACA |  |
|  |  | CCTTGTAGTCCATTTGTT **GGATCC** CTA CTCTATGACGTCGCTCATCG |  |
|  | cLUC-ZmWAKL^Y/362-630^ | TACGCGTCCCGGGGC **GGTACC** AGAGAAAAGCTCGGACAAGG |  |
|  |  | CCTTGTAGTCCATTTGTT **GGATCC** CTA CTCTATGACGTCGCTCATCG |  |
|  | cLUC-ZmWAKL^Q/340-666^ | TACGCGTCCCGGGGC **GGTACC** TGGGGTCTAAAATCTGCACA |  |
|  |  | CCTTGTAGTCCATTTGTT **GGATCC** CTA TATGACCTCACTCATCGTCT |  |
| qRT-PCR primers | *ZmPEX16* | CTCCAGAAATTCACGTCACATC | Zm00001d013212 |
|  |  | TATCTACTACGTGTGCGAAACT |  |
|  | *ZmPEX64* | CACTCTACCAACCTATGTGTCC | Zm00001d029747 |
|  |  | GCACTAGCTAGCTTTTATACGC |  |
|  | *ZmNPR1* | GTGTCTTGTGCTGACCCAGT | Zm00001d012660 |
|  |  | CCCTGAGTGGGAAGCAACTT |  |
|  | *ZmHRL* | CACGACCATGGGCTTTATCT | Zm00001d025020 |
|  |  | GTCCACCGTCATTTCCAAATTC |  |
| *ZmHRL* knock out plants screening primers | HRL-cas9 | AAGACCGACTCGACCAGACA |  |
|  |  | GCCGACGAACGAGATAAAGC |  |

**Table S3 The source data for all figures.**
